# Pacifier Sizing as a Prescription for Better Oral Health Outcomes for Infants: A Call to Action

**DOI:** 10.3390/children12091257

**Published:** 2025-09-18

**Authors:** David A. Tesini, Clive Friedman, Adithya Kethu, Kristin W. Hendricks

**Affiliations:** 1 Department of Pediatric Dentistry, Tufts University School of Dental Medicine, Boston, MA 02111, USA; 2 Pediatric Dentistry/Orthodontic Practice, Natick, MA 017601, USA; 3 Toothprints Inc., Hopkinton, MA 01748, USA; kethuadithya3389@gmail.com; 4 Dentistry Department of Pediatric Dentistry, Schulich School of Medicine, London, ON N6A5C1, Canada; clivesf@uwo.ca; 5 Private Pediatric Dentistry Practice, London, ON N5X3W3, Canada; 6 Private Pediatric Dental Practice, Santa Fe, NM 87505, USA; drhendricksdds@gmail.com; 7 Therapy Associates-Pediatric PT, OT, SLP, Santa Fe, NM 87505, USA

**Keywords:** pacifier, pacifier size, finite element analysis, growth and development, pacifier impact, oral health of infant

## Abstract

Sucking is essential for feeding and impacts the development of the cranio-facial-respiratory complex (CFRC). Non-nutritive sucking on a pacifier causes palatal narrowing and modifies the natural balanced relationship between intraoral pressure, peristaltic action of the tongue and the palate. Advanced engineering models have shown that malocclusions caused by pacifier use, are often a result of improper sizing. The sizing of pacifiers has historically been based on chronological age. *Chronological age is not a size metric*. Undersized pacifiers in a baby’s mouth can cause growth complications, palatal collapse airway incompetence and other orthodontic problems that can last a lifetime. Technical advances in facial anthropometrics and predictability of the rapid growth of the infant palate, can guide recommendations for pacifier size and design. This encourages change to a model of biometric sizing. Smartphone applications are being developed that use Ai and machine learning can predict conformity between palatal width and pacifier width.

## 1. Introduction

With a baby’s first breath, the cycle of suck, swallow, and breath begins. This reflexive drive, beginning in utero, is essential for feeding and impacts the development of the cranio-facial-respiratory complex (CFRC) [1]. The optimal development of the CFRC is multi-faceted and driven by a combination of neuronal networks, nasal breathing, tongue positioning, frenum attachments, muscle tone, breastfeeding, mandibular position, and free mandibular movement [1,2,3]. As it evolves, it includes TMJ development, chewing mechanics, and early tooth eruption [3]. The need for an infant to enhance soothing and feeding by using non-nutritive sucking (NNS) of digits and or pacifiers can likewise create disturbances that impact the CFRC [4,5,6,7]. Often unwanted narrowing of the palate or facial structures occurs and becomes a precursor to unhealthy airways [8]. This is often diagnosed in the presence of non-nutritive sucking with a poorly designed or improperly sized pacifier recommended by the infant’s chronological age [9]; *chronological age is not a size metric!*

Pediatricians, pediatric dentists, and other pediatric care providers often serve as first- line screeners and can identify these irregularities of growth and diagnose developing disharmonies of the CFRC. These are particularly maxillary transverse deficiencies with high vaulted, narrow palates. Clinical examination findings also discover other comorbidities such as anterior open bites, posterior crossbites, airway issues, OSA, sleep disordered breathing, other aberrant habits, baby weaning, and speech [7,10,11].

When the marketing of soothing and feeding products becomes involved, the best-intentioned parents, lactation consultants, doulas, nurses, and doctors can be easily confused and misled by manufacturer and social influencer narratives. Pacifiers are often advertised as being ‘good for oral development’ or ‘promoting healthy oral development’, but these types of claims are rarely supported by robust evidence-based science. While there is some research to suggest that some bulb shapes contribute to higher or lesser degrees of malocclusion, few companies produce research to support recommendations for their sizing stages based on chronological age [6,12]. The benefits of any given shape (orthodontic or traditional) become irrelevant when a pacifier is inappropriately sized for the infant/toddler [13]. Knowledge of anthropometrics and advancement in facial biometrics now allows us to recommend the proper size for each infant based on quantified measurable metrics not the variability inherent in the chronological age [9,14,15].

This descriptive methodology, proof of concept paper will focus on palatal growth, pacifier use, and current developments in technology, design and sizing of pacifiers that may impact the development of the cranio-facial-respiratory complex (CFRC).

### 1.1. The Cranio-Facial-Respiratory Complex (CFRC) and Palatal Growth

The cranio-facial respiratory complex (CFRC) describes the integrated anatomical and functional system comprising cranial bones, facial structures, fascial tissues, and the upper respiratory tract [1]. This complex plays a critical role in cranial motion, respiratory mechanics, structural balance, and neurological regulation. Key components include the maxilla, mandible, sphenoid, nasal passages, and associated soft tissues and fascial planes. The CFRC develops through both genetic programming and environmental stimuli such as prenatal positioning, birth mechanics, and postnatal functions—particularly breathing, sucking, swallowing, and oral posture.

Disruptions to this developmental trajectory—such as birth trauma, aberrant non-nutritive sucking (NNS), or poor oral posture—can result in downstream consequences. These include compromised airway patency, facial asymmetry, altered neuromotor function, sleep-disordered breathing, malocclusion, and temporomandibular joint (TMJ) dysfunction.

The maxillary arch undergoes rapid growth in the first two years of life, with the inter-canine and posterior widths increasing by over 5 mm [16,17,18,19,20,21,22]. Palatal surface width may increase by up to 8 mm during this time [20,23]. These changes are foundational for normal orofacial development, including occlusion, nasal breathing, and tongue posture—all components governed by the CFRC.

**Figure 1:**
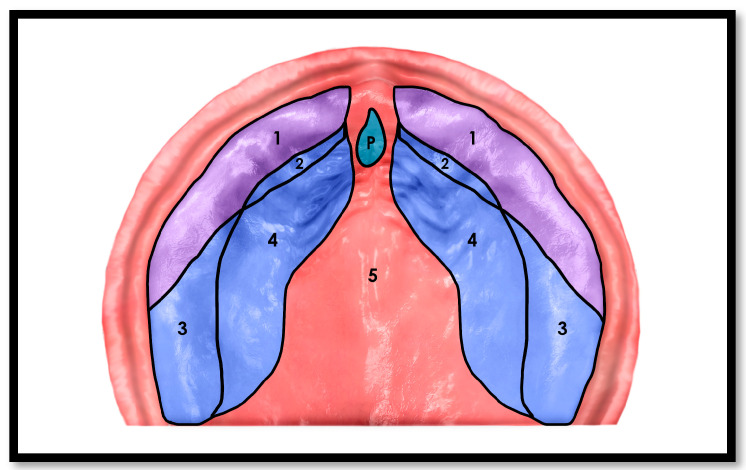
Pressure loading areas during NNS sucking. Newborn palatal anatomic areas adapted from [28]. 1,3 Dental Alveolar ridges; 2,4 Lateral Palatal Shelves (tektal wall); 5 Palatal Vault; *p* palatal foremen. Numbers refer to areas indicated on Illustration.

The natural pressure exerted by the tongue during suckling provides a crucial stimulus for maxillary growth by applying upward and lateral forces to the palatal shelves (tektal wall) (Figure 1). In contrast, pacifiers generate negative intraoral pressure and perioral compression, leading to transverse dental arch narrowing and anterior palate constriction [2,24,25,26]. Displacement of the tongue inferiorly by the pacifier bulb further inhibits this natural growth mechanism [27].

Emerging evidence links early palatal narrowing with upper airway restriction contributing to pediatric obstructive sleep apnea, and increased risk of sudden infant death syndrome [10,11,29]. Functional feeding and food progression—including chewing and baby-led weaning—also promotes balanced CFRC development.

### 1.2. Mandibular and TMJ Development

While attention often focuses on pacifier bulb design and the effect on palatal growth, the mandible and joint are also influenced by sucking habits. TMJ formation is driven by mandibular motion and masticatory muscle activity, especially following the eruption of the first primary molars around 16 months. Palatal width has also been shown to affect the position of the mandible [3,30]. Pacifiers must mimic physiological forces, particularly in size and shield design, to support normal CFRC growth.

**Figure 2:**
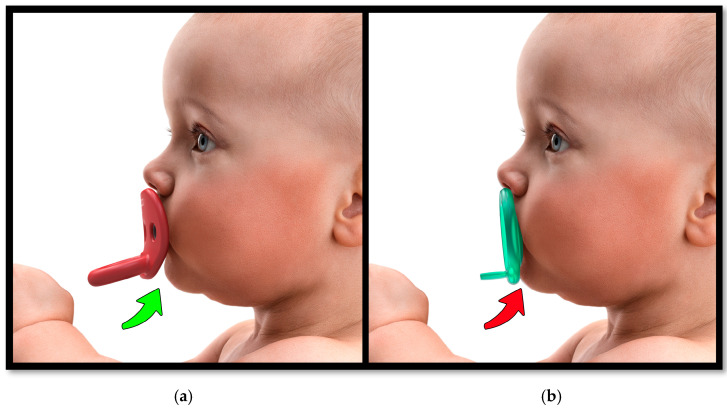
A biometric shield allows room for free mandibular jaw movement while sucking. (**a**) Biometric shield; (**b**) flat shield.

Restrictive shields—particularly those with vertically flat contours pressed tightly against the lips—can inhibit sagittal mandibular motion by exerting compressive forces during sucking. This mechanical ‘suck-back’ can trap the mandible, impeding normal forward growth. When pacifier shields allow free unobstructed mandibular movement, they may offer protective effects on airway development [8] (Figure 2).

### 1.3. Malocclusion and Non-Nutritive Sucking

The most prevalent malocclusions associated with pacifier use are anterior open bites (AOB) and posterior functional crossbites (PFC) [4,5]. Additionally, Overbite, Overjet, and Class II primary molars and canines are all viewed in the context of duration, frequency, and intensity of use. Together, they create complex malocclusions. While AOB often resolves after cessation of the habit, PFC (posterior functional crossbites) tends to persist [31,32]. Palatal collapse and crossbites are not self-correcting [33] and their severity correlates with the frequency, duration, and intensity of NNS. Although Caleza-Jiménez 2024 has reported a reduction in malocclusion with physiologic orthodontic pacifier shapes [12], a systematic review of malocclusions reported no significant differences between the orthodontic and conventional designs on the implications for malocclusions [6]. A likely explanation for this is that inappropriate sizing negates the functionality of differing bulb designs when in the palate. In a recent scoping review Hung et al. (2025) presented a very comprehensive and all-encompassing review of pacifier use and its influence on pediatric malocclusions [7].

Finite element analysis (FEA) biomechanical models now provide detailed insights into stress distribution during pacifier use [34]. FEA models simulate interactions between pacifier design and intraoral pressures, revealing that poorly sized pacifiers cannot always provide the palatal support offered by the tongue and breast [13,34,35,36,37,38,39,40]. This is creating a shift to evidence-based designs as bulb designs should therefore be adapted to growth-related changes in the jaw. These biomechanical models give us the insight needed to understand how pacifier sizing and design cause clinical effects. They allow us to investigate the effects that size, geometry relative positions, loads, and materials have on the effect of the pacifier’s bulb contact with palate, teeth, and tongue. It helps us to understand how pacifiers cause changes in the CFRC.

### 1.4. The Pacifier Paradox: Design, Sizing, and Development

Retail pacifier sizing recommendations are based on chronological age stages (e.g., 0–3 months, 6–12 mo., 6 mo.+, 0–9 mo., 6–18 mo.), but there is no industry standard for defining these stages. Inconsistencies exist within brands and between brands with pacifier widths ranging from 12.1 mm to over 25 mm for the same age categories (Table 1).

**Figure 3:**
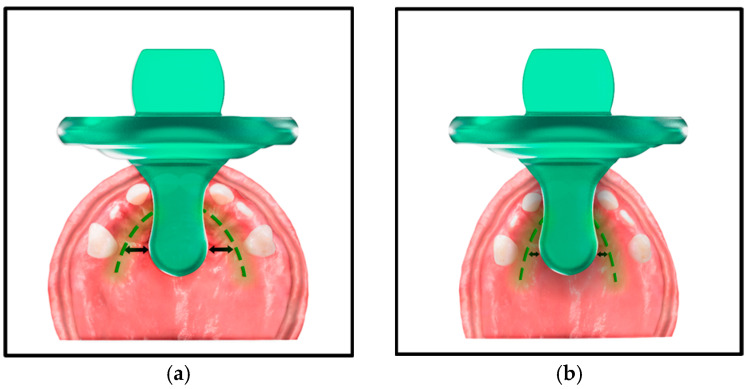
Illustrated example of a “too small” pacifier in the palate of a 14-month growing infant showing: (**a**) lack of palatal support (**b**) the resulting loss of palatal width due to the lack of palatal support caused by a “too small” pacifier.

A prerequisite for an anatomically correct pacifier is that it corresponds to the natural growth process of the child’s palate. Recently, Sistenich et al., 2022 studied 77 pacifiers/13 brands and concluded that pacifiers are too undersized in width, length, and height to physiologically fit the palate structures [40]. The physiologically aligned pacifiers studied did not achieve the age-specific dimensions of the palate; they are usually sized too small (Figure 3). Brand recommendations of age-specific conformity of shape and size between commercial pacifiers and palates of infants could not be validated [40]. Chronological age sizing does not present consistent recommendations across brands or within brands and this usually results in under sizing [40] (Figure 3). Statements made in a recent review, encouraging that a pacifier should be as small as possible, cannot be supported [12]. Likewise, package marketing that states ‘for ages 0 + months’ must surely be confusing to parents and caregivers.

Further, the marketing claims that emphasize ‘orthodontic’, ‘jaw-fitting’, ‘healthy for natural oral development’, ‘anatomically correct’ or ‘Good for oral Development’ are not held to a standard made available with the use of advanced FEA engineering models [13,34]. These engineering models validate the need for proper sizing for pacifier fit.

### 1.5. Evolving Technology for Improving Oral Health and Wellness Outcomes

The future of pacifier design and infant oral care lies in leveraging engineering tools, anthropometric data, and facial biometrics to guide sizing and design selection. This shift to biometric sizing allows manufacturers, clinicians, and caregivers to move beyond non-standardized, random age guidelines and instead tailor pacifier choice to each infant’s unique craniofacial morphology. Such individualized approaches may mitigate the risk of interfering with CFRC development. The recent Academy of Pediatric Dentistry (AAPD) *Policy on Pacifier Use* comments that the use of biometrics to aid pacifier selection has shown promise in recent research [41].

With the current lack of conformity between manufacturers, a new method based on biometric sizing has been proposed. A palatal width/pacifier size biometric has been described [9]. This is possible by using available data published on palatal widths and applying a fitted linear algebraic model to calculate the proper pacifier width and stage [20,21,42,43] (Electronic Supplemental Data S2).

**Figure 4:**
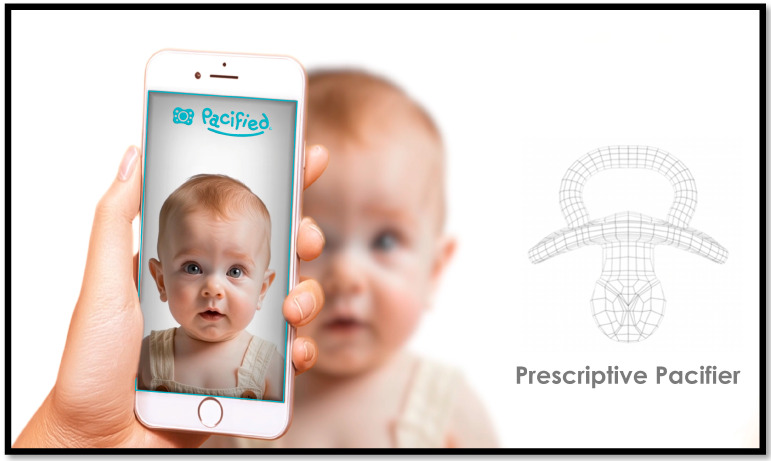
A simple oriented photograph from a smartphone allows facial recognition technology to biometrically locate anthropometric landmarks.

**Figure 5:**
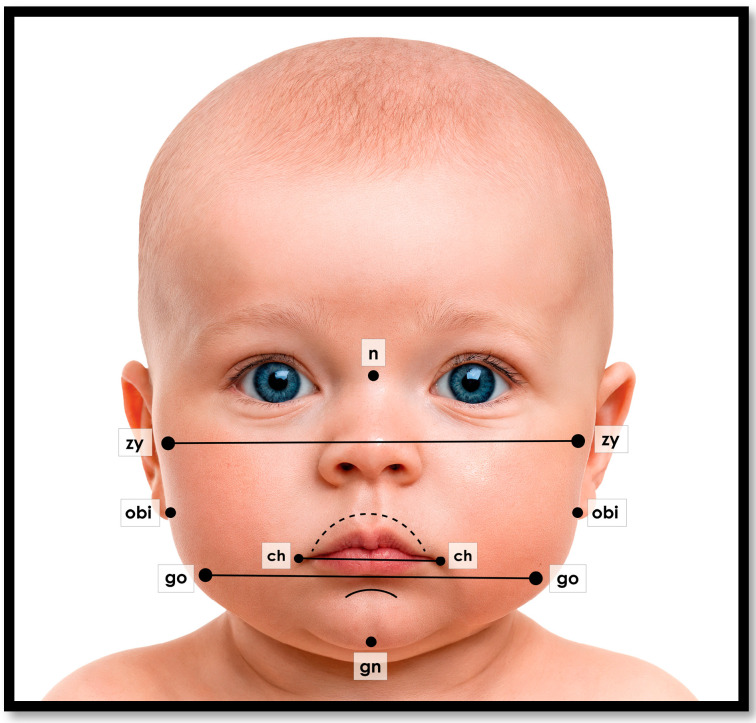
Examples of anthropometric facial landmarks captured from an oriented facial photograph. Intermolar palatal width (from facial proportions), mouth width (cheilion–cheilion), mandibular width (gonion–gonion), facial width (zygion–zygion), Otobasion inferius (OBI), soft tissue nasion (N), and soft tissue gnathion (GN). The palate is indicated by the hashed line arch.

This biometric sizing model has been implemented in the smartphone technology of the first-generation *Pacified^®^ app*. This application uses facial recognition and anthropometric analysis within a machine learning-based random forest algorithm. It can recommend pacifier size and shield design from a single-oriented photograph (Figure 4). Key biometric landmarks are captured by facial recognition programming (Figure 5).

Innovative smartphone applications offer a simple, scientific, yet intuitive way to find the right size pacifier for the infant. A weighted algorithm uses parametrics based on these well-defined anthropometric landmarks, published facial proportions, and demographic data [14,15].

Additionally, mandibular size and position can be defined by the Mandibular Index of Horn, based on additional anthropometric landmarks of otobasion inferius (OBI), soft tissue nasion (N), and soft tissue gnathion (GN) [29]. This provides concurrent information for improving designs of the pacifier shield that allow for free mandibular movement (Figure 2). This particular index is important for infants with small jaws, mandibular retrognathia, and those with airway insufficiency.

The facial anthropometrics, facial correlations, mandibular index, and demographic data (age, weight, sex) are then integrated into a predictive algorithm that uses metric facial characteristics within a machine learning-based random forest algorithm. These data can then be input into a dataset of commercially available pacifiers through the *Pacified^®^* app as brands develop their own smartphone platforms. A biometrically sized pacifier, specific to the baby’s own anatomy, is then recommended from this dataset to provide parents with a scientifically validated *size*/*fit standard* (Electronic Supplemental Data S2).

Because manufacturers design pacifiers with baby wellness in mind, they need a model to transition from chronological age to biometric sizing. This was accomplished by using the Range Rule) [44] (Electronic Supplemental Data S2). It provides brands defined biometric stages for incorporating their pacifier SKUs (Stock Keeping Units) (Table 2) This model serves as a quantitative tool for recommending suitable pacifier widths based on palate size. The millimeter widths of pacifiers can simply be added to the marketing and packaging. Biometric sizing will encourage transition to a healthcare focused model for retailing of pacifiers. It will encourage research and education of dental, medical, and allied health professionals to reduce adverse effects of pacifier use on the development of the CFRC.

Progressive data acquisition will populate smartphone and software programs that leverage palatal–pacifier correlations. Machine learning and AI applications that are now being developed, will increase predictability by using growth models to inform parents when to transition to the next biometric pacifier size. With the availability of advanced engineering, evolving smartphone technology, machine learning algorithms, and interpretive AI, we can prescriptively participate in the advancement of baby/toddler oral health and wellness outcomes.

It is time to rethink beliefs that chronological age is a size metric… *it is not.*

## 2. Conclusions

1. The harmful effects of pacifier use must be recognized within the context of the development of the CFRC and evaluated by the effects of long-term Oral Health and Wellness Outcomes.
2. Modern engineering models should be used by the baby product industry to evaluate the functional aspects of pacifiers.
3. Biometric sizing represents a potentially significant development in pediatric oral care. As AI and machine learning continue to evolve, predictive models may soon inform not only pacifier selection but also the optimal timing for upsizing based on projected palatal growth trajectories.
4. Advancing technology needs to be leveraged in longitudinal clinical validation studies.

## Figures and Tables

**Table 1:** Inconsistent metric and chronological age descriptors of selected pacifiers as recommended by brands (Electronic Supplemental Data S1).

Chronological Age	Biometric Width (mm)	Brand/Pacifier Trade Name
0–3 mo.	12.5	Philips Avent *Soothie*
3–18 mo.	12.5	Philips Avent *Super Soothie*
0–3 mo.	18.7	MAM *Original Start*
0–3 mo.	15.4	MAM *Comfort*
0–6 mo.	17.6	NUK *Orthodontic*
18–36 mo.	23.1	NUK *Orthodontic*
0–12 mo.	17.3	NUK *Nature Comfy Duet*
18–36 mo.	17.6	Tommee Tippee *Nighttime Orthodontic*
0–6 mo.	12.9	Dr Brown *Happy Paci*
0+ mo.	19.0	Ryan & Rose *Cuttie Pat Smile*
0–6 mo.	12.1	Itzy Ritzy *Natural Rubber Soother*

**Table 2:** Biometric sizing metrics for the pacifier retail market (Electronic Supplemental Data S2).

Biometric Staging with Overlap Based on Range Rule ([44])		
Newborn	≥12.0 mm	≤14.0 mm
Biometric Stage 1	≥13.3 mm	≤16.4 mm
Biometric stage 2	≥15.7 mm	≤20.9 mm
Biometric stage 3	≥18.9 mm	≤25.0 mm

## Data Availability

Data will be made available upon reasonable request to corresponding author.

## Supplementary Materials

The following supporting information can be downloaded at https://www.mdpi.com/article/10.3390/children12091257/s1, Electronic Supplemental Data S1: Pacifier Digital Measurement Methodology. Electronic Supplemental Data S2: Mathematical Model: Using a data driven approach to biometrically sizing pacifiers.
