# Supplementary material for: Pacifier Sizing as a Prescription for Better Oral Health Outcomes for Infants: A Call to Action
Source: Children (Basel). 2025 Sep 18;12(9):1257. doi: 10.3390/children12091257 (PMC12469228; doi:10.3390/children12091257)
Supplement: Supplementary file 1 [file children-12-01257-s001.zip › children-3842425 Supplemental change.pdf]

Supplementary Materials:

Electronic Supplemental Data S1

Pacifier Digital Measurement Methodology

Qualitative manufacturing information of the metric sizes pacifiers marketed in the USA is not available. This information is important for proper pacifier selection.

For the purpose of this paper, twenty-eight widely distributed pacifiers were purchased from a combination of online retailers (Amazon.com) and a big US box store (Walmart) in May 2025. The pacifiers were sorted by brand and age recommendations as listed on the packaging and their respective websites.

Two dental assistants were trained in the use of a General Carbon Fiber UltraTech digital caliper. They were individually instructed to measure each pacifier for length from the base to tip of nipple at the contact point of the shield, and the width at the widest point of the nipple bulb. The caliper was calibrated between each measurement.

The first dental assistant measured the pacifiers and collected the data. The second dental assistant was then asked to do the same. The assistants were blinded to the results of the other.

The data was then collected, and an average length and maximum width was determined for each pacifier brand and type. Requests were sent to the individual brands for verification of the measurements, but as of this writing, none have agreed to provide verification data. *(see Table 1 main manuscript)*

Electronic Supplemental Data S2:

Mathematical Model: Using a data driven approach to biometrically sizing pacifiers

Quantitative data on the width of the palates of infants and toddlers are readily available as referenced in the manuscript [20,21,40,42,43]. Peyton (1931) [20] reported the maximum transverse palatal width across the alveolar ridge, actual surface width of the palate, along with the corresponding age, weight and gender, important for establishing consistent anatomical correlations. Likewise, biometric pacifier widths (mm) are available by direct measurement of commercially available pacifiers. However, when investigating the relationship between Palate Width and Pacifier Width., no quantified data was available from any reported manufacturer's studies. A mathematical, exploratory/proof of concept.model was developed

The lack of an evidence-based correlation between Palate Width and Pacifier Width presents a significant challenge in designing and recommending pacifiers that are appropriately sized for the dynamic anatomical growth of the infant palate. Research in infant palatal measurements has variations in methodology, including measurements taken from canine to canine, tuberosity to tuberosity,

molar to molar and maximum arch width. Awareness to these variations must be considered.

To establish this quantitative relationship between palatal width and pacifier nipple width, we developed a predictive model. We derived five representative data points from Sistenich et al Figure S1 [40], which illustrates box-plot trends of infant palatal width and pacifier nipple width across age quarters (0-2, 2-4, 4-6, 6-11, and 11-14 months). Using the matplotlib library in Python, we manually digitized the figure by recording coordinate points, allowing us to estimate the approximate mean values of palatal width (PW) and pacifier nipple width (NPW) across each age quarter. These five data points served as the foundation for an extrapolated dataset of 1000 points, generated by incorporating natural variance consistent with the distributions observed in the original figure. We then plotted this comprehensive dataset on a scatter plot (*Figure S1*). Through rigorous regression analysis of these 1000 data points, we identified the best-fit linear model that accurately describes the relationship between pacifier nipple width (Y) and palatal width (X), where palatal width is defined as the widest transverse distance across the alveolar ridge.

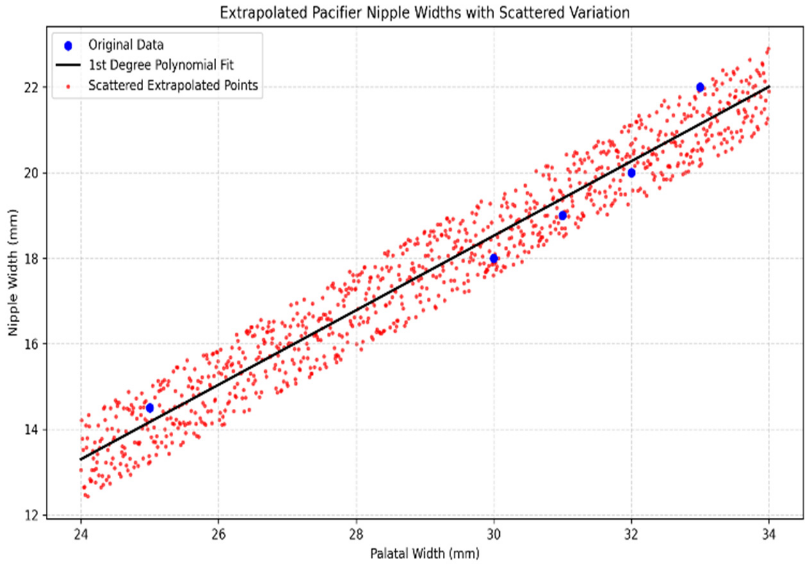

Figure S1. Extrapolated Pacifier Nipple Widths and Palatal Widths using data from Sistenich et al 2022 (Adapted from [40]).

Fitted Linear Model

$$Y = 0.8711 * X - 7.6082$$

OR

$$X = (Y + 7.6082)/0.8711$$

This linear model demonstrates an excellent fit ($R^2 \approx 0.995$), indicating that palatal width (X) accounts for nearly all of the variance in pacifier nipple width.

For instance, if Palatal width (X) = 32.0 mm: then the nipple width (Y) = 20 mm as calculated from the sourced data with 94.1% accuracy

$$Y = 0.8711 * 32.0 - 7.6082 \approx 20.267mm$$

To ensure robust validation of the proposed pacifier-palatal width correlation, a comprehensive developmental dataset was first

synthesized. We aggregated foundational data—specifically infant age and corresponding palatal widths—from several seminal studies, including Peyton (1931), Hohoff et al. (2006), Kihara et al. (2017), and Bruggink et al. (2019) [20,21,42,43].

Initial analysis revealed that this aggregated dataset had an unequal distribution of observations across the different biometric size categories. To correct this imbalance and create an unbiased validation set, we expanded the dataset to 10,000 data points using a *balanced-class rectangle-uniform extrapolation* technique. The procedure was as follows:

1. **Identification of Boundaries:** We first identified all under-represented size categories within the source data. For each of these categories, we established its precise clinical boundaries—the minimum and maximum values for both infant age and palate width.
2. **Constrained Data Generation:** We then generated new, synthetic data points for each sparse category. This was achieved by randomly sampling age-and-width data pairs from a uniform distribution that was strictly constrained within the pre-defined boundaries for that class, ensuring all new data was biologically plausible.
3. **Dataset Balancing:** This sampling process was iterated until every size category was fully populated, achieving a target of exactly 2,500 observations per class. This step ensures equal representation across all categories, eliminating potential model bias. The entire procedure utilized a fixed random seed to guarantee full reproducibility.

This enlarged and harmonized dataset served as a robust benchmark for testing. Finally, our pacifier-palatal linear equation was applied to each of the 10,000 entries to calculate a predicted pacifier width. These predictions were subsequently validated against the extensive data taken from the scientific literature to ensure they were mathematically valid, by comparing them to the ideal bulb width ranges established for each biometric stage (Table S1(a)).

This validation process demonstrated a 94.1% accuracy, indicating that the predicted pacifier widths correctly corresponded to the appropriate anatomical stage. This result substantiates the model's efficacy as a quantitative tool for estimating anatomically suitable pacifier widths from direct palatal measurements, offering a more precise, individualized alternative to the traditional age-based sizing.

With the model's predictive accuracy established, the focus shifted to its operationalization for industry use. This involved developing the comprehensive standard (Table S1)by first addressing the current use of chronological age-based pacifier sizing. We measured 74 commercially available pacifiers across 24 distinct brands and systematically mapped them onto our biometric sizing chart. Method of measurement consistent with description in Supplemental Data S1. To account for the inherent deficiencies and variability in chronological age staging among manufacturers, we employed the *"range rule,"* a statistical approach that incorporates standard deviations [44]. This rule ensures that approximately 95% of the data falls within two standard deviations of the mean, providing the necessary flexibility for brand compliance in their marketing strategies. This robust descriptive

data, combined with our established biometric model, culminates in a scientifically validated Standard Sizing/Fit presented within our new biometric (*Table S1*).

Table S1. Mathematical model applied for determining pacifier size /palatal width relationship. (a) Palatal width size ranges (mm) (b) Biometric Pacifier Sizing Stages (mm).

**Stage**	**Palatal Width Size Range (mm)**	**Biometric Staging with overlap based on Range Rule ([44])**		
Newborn	23.5–28.5 mm	Newborn	≥ 12.0 mm	≤ 14.0 mm
Stage 1	24.0–27.3 mm	Biometric Stage 1	≥ 13.3 mm	≤ 16.4 mm
Stage 2	26.0–28.9 mm	Biometric stage 2	≥ 15.7 mm	≤ 20.9 mm
Stage 3	28.0–34.5 mm	Biometric stage 3	≥ 18.9 mm	≤ 25.0 mm

(a) *(b)*

The future of biometric sizing for infant pacifiers holds significant promise. Our ongoing work involves a robust Random Forest model that leverages this palatal-pacifier correlation to recommend when an infant can transition to the next pacifier size (Newborn to Stage 1, Stage 1 to Stage 2, and Stage 2 to Stage 3). While the linear polynomial presented here offers a powerful "tool”, that will evolve as further longitudinal data is collected. Continuous data acquisition will enhance the predictive power of our Ai models, leading to anatomically appropriate pacifier recommendations for infants/toddlers.

Limitations should be noted in that only one study provided anatomical correlations between Palate Width and Pacifier Width [40]. The deficiency of evidence-based–correlations between Palate Width and Pacifier Width will be resolved as progressive clinical data becomes available. Recommending pacifiers that are appropriately sized for the *dynamic* anatomical growth of the infant palate remains a forward looking objective. It should be noted that available reference sources on palatal width can present methodological variations, between the measurement criteria used in Peyton’s 1931 report and those used in more contemporary studies. Attention should be given to this when using these resources.
